# A Rare Case of Xanthogranulomatous Cystitis Mimicking Bladder Malignancy in Nigeria: A Case Report and Review of Literature

**DOI:** 10.7759/cureus.37196

**Published:** 2023-04-06

**Authors:** Onyekachi C Nwokoro, Francis I Ukekwe, Chika S Chijioke

**Affiliations:** 1 Department of Morbid Anatomy, University of Nigeria, Enugu Campus/University of Nigeria Teaching Hospital, Enugu, NGA; 2 Department of Morbid Anatomy, University of Nigeria Teaching Hospital, Enugu, NGA

**Keywords:** mimic, histopathology, urinary bladder, cystitis, xanthogranulomatous

## Abstract

Xanthogranulomatous cystitis (XC) is a very rare urinary bladder condition, of unknown etiology. It may mimic bladder malignancy; therefore, histopathologic assessment is crucial in diagnosis. We report a case of a 38-year-old female who presented with persistent, painless hematuria and a strong consideration of bladder malignancy clinically and on cystoscopy. However, on histopathologic evaluation, the rare diagnosis of XC was made. She received a course of antibiotics and has remained asymptomatic after four months of follow-up. To the best of our knowledge, this is the first reported case of XC in Nigeria and Africa.

## Introduction

Xanthogranulomatous cystitis (XC) is an extremely rare, benign disease of the urinary bladder of unclear etiology [1-3]. Although xanthogranulomatous inflammatory changes occur commonly in other sites, such as kidneys, gall bladder, salivary glands, ovaries, endometrium, pancreas, brain, colon, and appendix, there are very few reported cases in the urinary bladder [2,4]. In the urinary tract, xanthogranulomatous pyelonephritis is commonly encountered; however, involvement of the ureters, bladder, and urethra by xanthogranulomatous inflammation is an extreme rarity [5].

Clinically and on cystoscopy, XC may mimic a bladder malignancy [1,6]. Other rare forms of cystitis which may also mimic malignancy clinically and on imaging include eosinophilic cystitis and florid cystitis cystica et glandularis [7,8]. These conditions, though benign, may present with solid masses in the bladder, with worrisome features on imaging, and prove quite challenging to diagnose clinically and radiologically, in the absence of histopathologic assessment [1]. Therefore, relying on clinical evaluation and radiological imaging only would not suffice for accurate diagnosis and the exclusion of malignancy [1,6].

XC is, indeed, extremely rare, and less than 40 cases have been reported in world literature since the first report in 1932 [1,9]. In this report, we describe a case of XC, mimicking bladder malignancy, in an adult Nigerian female. To our knowledge, following a thorough medical literature search, this is the 39th reported case of XC in world literature and the first reported case in Nigeria and Africa generally.

## Case presentation

A 38-year-old African (Nigerian) female presented to the urology clinic with primary complaints of persistent hematuria of three months' duration. She was moderately pale on physical examination, with a hemoglobin concentration of 9.6 g/dl, but afebrile and anicteric. Her blood pressure was 110/70 mmHg. The total white blood cell (WBC) count (9.0 x 10^9^/L) and differentials, platelet count, serum electrolytes, urea and creatinine, and liver function parameters were all within normal reference ranges. She had received more than 10 units of blood on account of the persistent gross hematuria.

There was a history of live birth by cesarean section four months prior to the presentation. There was no other significant past medical history. Cystoscopy showed a pulsating bladder mass on the right bladder wall.

A transurethral resection was done, with an uncertain histopathologic diagnosis. The hematuria persisted, and a bladder malignancy was strongly suspected clinically. Therefore, an open resection was done, during which a 4.0 cm x 3.0 cm bladder mass was found and completely resected.

**Figure 1:**
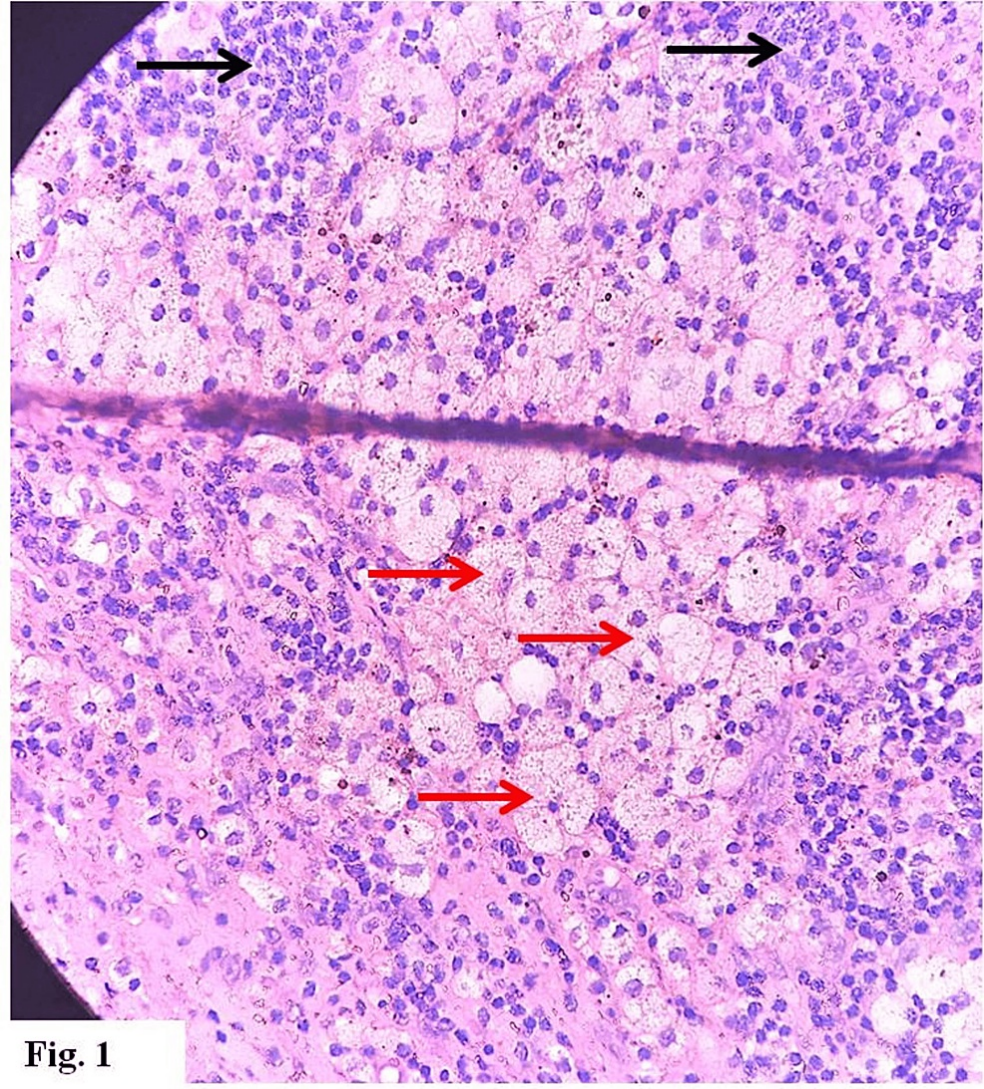
Numerous lipid-laden macrophages (red arrows) and lymphocytes (black arrows) (hematoxylin & eosin stain x 400)

**Figure 2:**
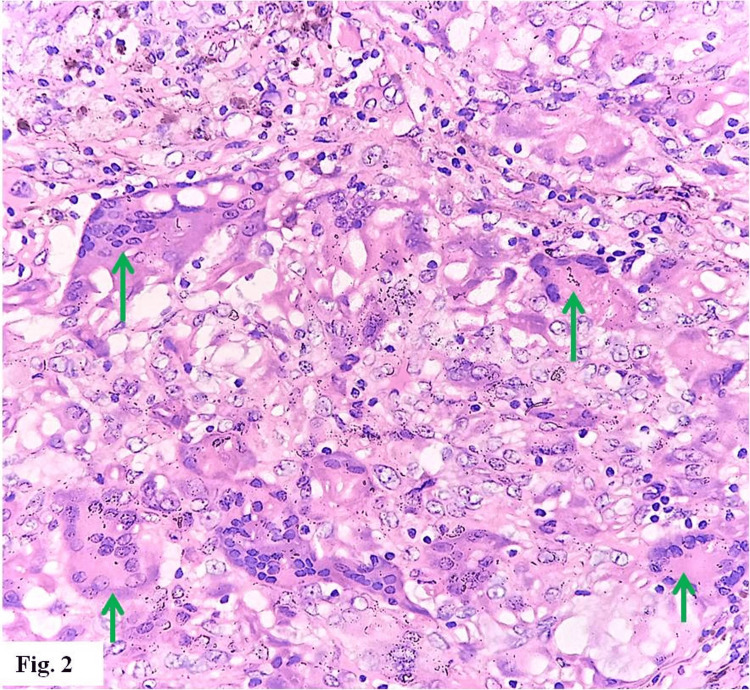
Histiocytic multinucleated giant cells (green arrows) (hematoxylin & eosin stain x 400)

**Figure 3:**
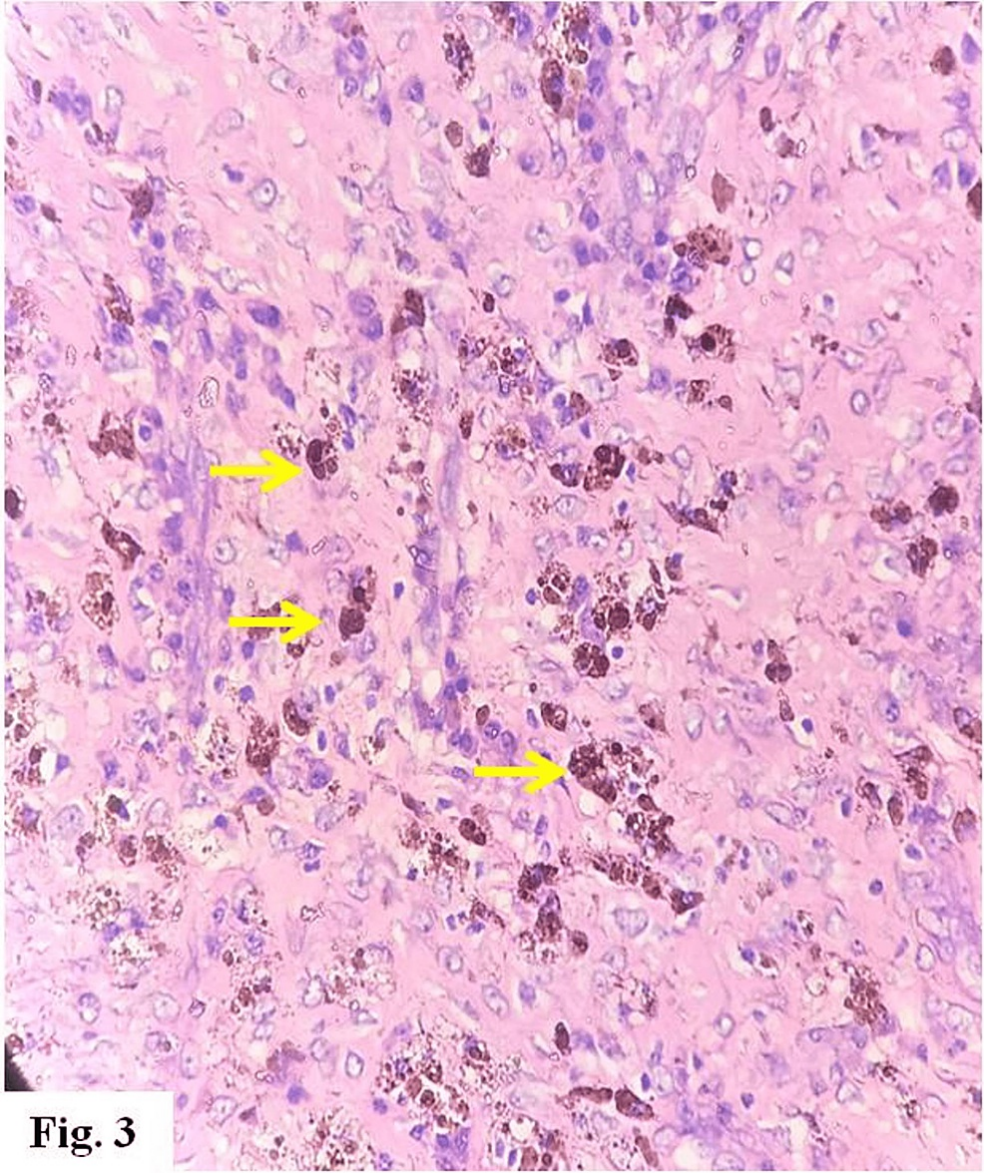
Siderophages (yellow arrows) (hematoxylin & eosin x 400)

**Figure 4:**
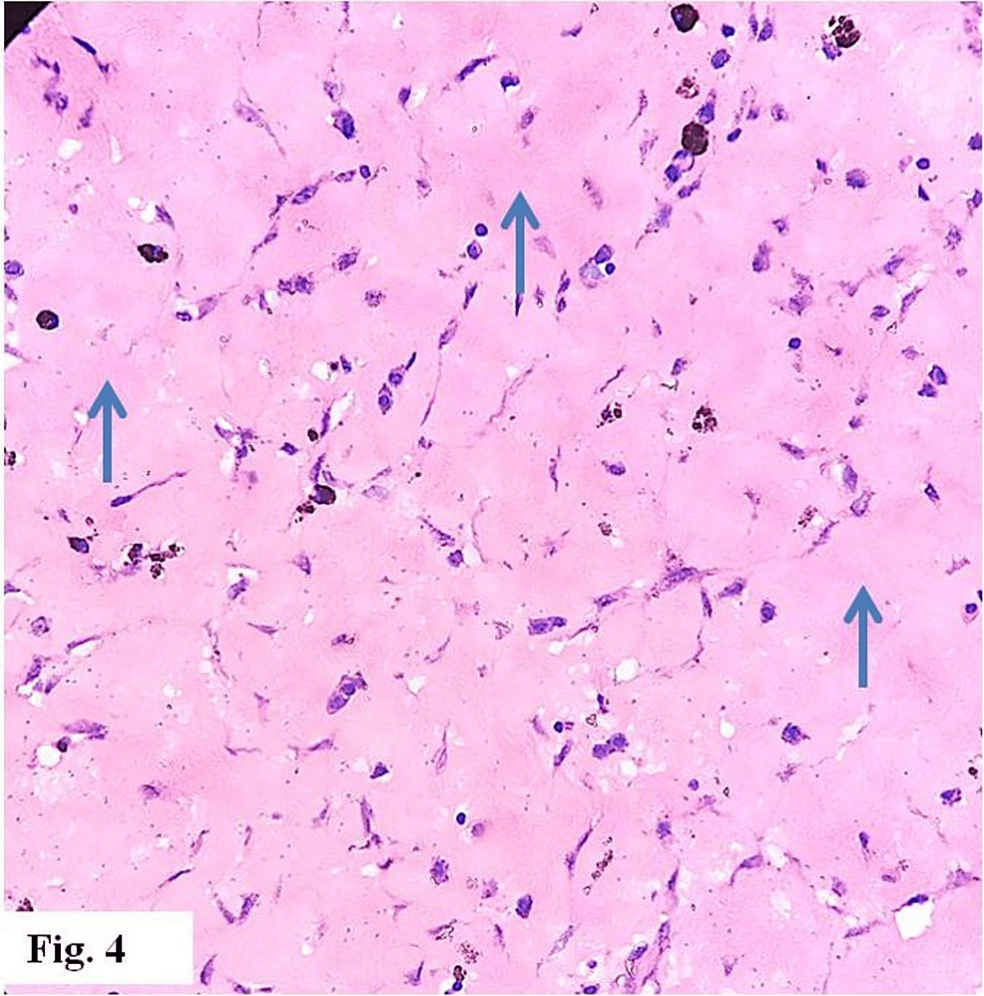
Areas of dense fibrosis (blue arrows) (hematoxylin & eosin x 400)

Microscopic histopathology of the resected mass revealed a chronic xanthogranulomatous lesion, with numerous lipid-laden macrophages; lymphocytes, plasma cells, and multinucleated giant cells were also present, with foci of fibrosis and siderophages (Figures 1-4). There was no evidence of malignancy.

A histopathologic diagnosis of XC was made. The patient then received intravenous antibiotics (levofloxacin and metronidazole) for the first 72 hours, followed by oral antibiotics (levofloxacin and metronidazole), hematinics, and multivitamin tablets for four weeks.

She had a stable and uneventful postoperative period. At the follow-up, four months later, there was no recurrence, and the patient remained completely asymptomatic.

## Discussion

XC is an extremely rare chronic granulomatous disease, with less than 40 reported cases in world literature since the first report by Wassiljew in 1932 [1,9,10]. This is the 39th report in the literature and the first in Nigeria and Africa.

The median age of all reported cases is 43 years, with a range of 4-76 years and a slight female preponderance (21 females, 17 males, 1 unknown gender) [1]. We have included this report on a 38-year-old female in the overall gender distribution.

The etiology of XC is still uncertain; however, various theories have been suggested, such as chronic urinary tract infections, altered lipid metabolism, autoimmune mechanisms, induction by a primary tumor or foreign body, and arising from urachal remnants or adenoma [1,3,10,11,12].

The majority of the reported cases (53%) involved the dome of the bladder, and 70% were associated with an abnormality of the urachus; involvement of the lateral, anterior, and posterior walls was less frequent [1,11,13]. We, however, found the lesion in this case to involve the right lateral wall, a less common site, which also makes association with the urachus unlikely.

XC may mimic bladder malignancy clinically and on imaging [4,6,14]. Although XC may mimic a malignant bladder lesion, it may also be associated with such malignant tumors [15]. The most frequent presenting symptoms are lower abdominal pain with a palpable mass, frequency, dysuria, and hematuria [4,11,13]. XC has no specific presenting symptoms, with a similar presentation as for bladder malignancy, constituting a clinical diagnostic challenge; furthermore, the possible presentation as a bladder mass may pose a challenge in radiological diagnosis, with strong consideration for bladder malignancy [1,6]. The patient in our report presented with persistent hematuria as a sole complaint and a pulsatile mass on the right lateral bladder wall on cystoscopy. In view of the clinical and radiologic diagnostic challenges, histopathologic assessment is very crucial for the accurate diagnosis of XC and the determination of the presence or absence of any bladder malignancy [1,6].

The histopathologic features of XC are foamy (lipid-laden) macrophages with multinucleated giant cells and mixed inflammatory cells, including lymphocytes, plasma cells, and neutrophils [1,3]. Although the diagnosis of XC on histopathologic evaluation is usually straightforward, certain differential diagnoses should also be considered and ruled out, such as malakoplakia, eosinophilic cystitis, and florid cystitis cystica et glandularis [7,8,15]. Malakoplakia involving the bladder shows similar morphologic features as described for XC but includes the presence of Michaelis-Gutmann bodies (iron-containing, cytoplasmic-laminated concretions) [15]. In eosinophilic cystitis, the inflammatory infiltrate includes prominent eosinophils within the lamina propria and muscularis or all layers of the bladder wall, with peripheral eosinophilia in some patients [7]. Cystitis cystica et glandularis is a common reactive change often seen in a background of chronic mucosal irritation or inflammation; when florid, it may appear as a mass on cystoscopy and may mimic malignancy [8]. On histopathologic evaluation, non-infiltrative, cystically dilated von Brunn nests with gland-like lumina and/or cystic cavities are readily identified on the inflammatory background [8].

The treatment of choice for XC is yet unclear, likely due to the rarity of the lesion, and the reported patients have had various management modalities, including simple excision, transurethral resection of bladder tumor (TURBT), and partial and radical cystectomy [1,3,14].

Medical treatment with broad-spectrum antibiotics has often been added following the histopathologic diagnosis of XC [3,4,14]. The index patient initially had a transurethral resection, followed by an open resection with a course of antibiotics for four weeks.

## Conclusions

XC is an extremely rare, benign lesion, which may mimic bladder malignancy and present clinical and radiologic diagnostic challenges. Histopathologic assessment is crucial to confirm the diagnosis. Due to the rarity of this condition, there is no consensus yet on the management guidelines, and current treatment modalities include surgical management, in addition to a course of antibiotics. This is a novel report of XC in Nigeria and Africa.
